# A Fluid Recommended as a Preservative from Syphilis

**Published:** 1851-12

**Authors:** 


*A fluid recommended as a Preservative from Syphilis.*—M. LANGLEBERT lately stated to the Academy of Medicine of Paris, that he had discovered a compound which, judging from the success of his experiments, would effectually preserve from syphilis. The following is the formula: Alcohol, one ounce and two and a half drachms; soft soap with stripes and prepared with potash, the same quantity; dissolve and strain, then add essential oil of lemon, five drachms. M. Langlebert mentioned the following experiment :—

He took purulent matter from a chancre with a hard base, and inoculated the left thigh of one of his pupils, who had volunteered his services. On the other thigh the inoculation was performed in a different manner: the lancet dipped in the same pus was made to scrape the skin and make it *raw*, so as fully to ensure absorption; after five or six minutes the preservative fluid was applied on this right side, and this was repeated three or four times. The usual effect was the next day perceived on the left thigh, but the right, where the prophylactic fluid had been applied, presented only a thin and dry crust. On the third day, the pustule on the left thigh was caüterized with strong nitric acid. A public experiment has since been made upon two other pupils, who had requested the *favor*, as well as upon M. Langlebert himself, and the success was complete. M. Ricord is to report upon this new prophylactic.—*London Lancet.*

